# Arousal Effects on Pupil Size, Heart Rate, and Skin Conductance in an Emotional Face Task

**DOI:** 10.3389/fneur.2018.01029

**Published:** 2018-12-03

**Authors:** Chin-An Wang, Talia Baird, Jeff Huang, Jonathan D. Coutinho, Donald C. Brien, Douglas P. Munoz

**Affiliations:** ^1^Centre for Neuroscience Studies, Queen's University, Kingston, ON, Canada; ^2^Graduate Institute of Humanities in Medicine, Taipei Medical University, Taipei, Taiwan; ^3^Research Center of Brain and Consciousness, Taipei Medical University, Shuang Ho Hospital, New Taipei City, Taiwan

**Keywords:** trial-by-trial, pupillometry, pupil dilation, parasympathetic and sympathetic system, locus ceruleus-norepinephrine

## Abstract

Arousal level changes constantly and it has a profound influence on performance during everyday activities. Fluctuations in arousal are regulated by the autonomic nervous system, which is mainly controlled by the balanced activity of the parasympathetic and sympathetic systems, commonly indexed by heart rate (HR) and galvanic skin response (GSR), respectively. Although a growing number of studies have used pupil size to indicate the level of arousal, research that directly examines the relationship between pupil size and HR or GSR is limited. The goal of this study was to understand how pupil size is modulated by autonomic arousal. Human participants fixated various emotional face stimuli, of which low-level visual properties were carefully controlled, while their pupil size, HR, GSR, and eye position were recorded simultaneously. We hypothesized that a positive correlation between pupil size and HR or GSR would be observed both before and after face presentation. Trial-by-trial positive correlations between pupil diameter and HR and GSR were found before face presentation, with larger pupil diameter observed on trials with higher HR or GSR. However, task-evoked pupil responses after face presentation only correlated with HR. Overall, these results demonstrated a trial-by-trial relationship between pupil size and HR or GSR, suggesting that pupil size can be used as an index for arousal level involuntarily regulated by the autonomic nervous system.

## Introduction

Physiological arousal constantly changes throughout the day, and this fluctuation greatly influences behavior and performance. Fluctuations in arousal are commonly linked to changes of the sympathetic and parasympathetic activity in the autonomic nervous system. Galvanic skin response (GSR) is an independent index of sympathetic activity while heart rate (HR) is predominantly controlled by the parasympathetic nervous system (1–4). The sympathetic nervous system controls sweat gland activity, and increases in sympathetic activity produce corresponding increases in GSR (5). Although HR is predominantly linked to the parasympathetic system and parasympathetic activation decreases HR, it is antagonistically controlled by both sympathetic and parasympathetic activity which can produce increased or decreased HR, respectively (3, 4).

Pupil size is also modulated by the balanced activity of the parasympathetic and sympathetic nervous systems (6, 7). Although an increasing number of studies have used pupil size to index the level of arousal (8–15), limited research has focused on examining the relationships between pupil size, HR and GSR. Nevertheless, many studies have concurrently recorded these measures to mostly investigate the task-dependent modulation in these physiological indexes [e.g., (16–22)]. Moreover, pupil size is modulated by low-level visual properties such as luminance, color, visual contrast, and spatial frequencies (23–26). Furthermore, eye movements influence not only the accuracy of pupil size measurement in the video-based eye tracking system, but also the parasympathetic and sympathetic activity via the pathway through the midbrain superior colliculus (27–30). Therefore, distinct patterns of eye movements in different conditions could influence pupil size differently via this pathway. Notably, in previous research, factors such as visual contrast, spatial frequency, color, and eye movements are usually not adequately controlled for and may have confounded observed effects between pupil size and arousal level.

**Figure 1:**
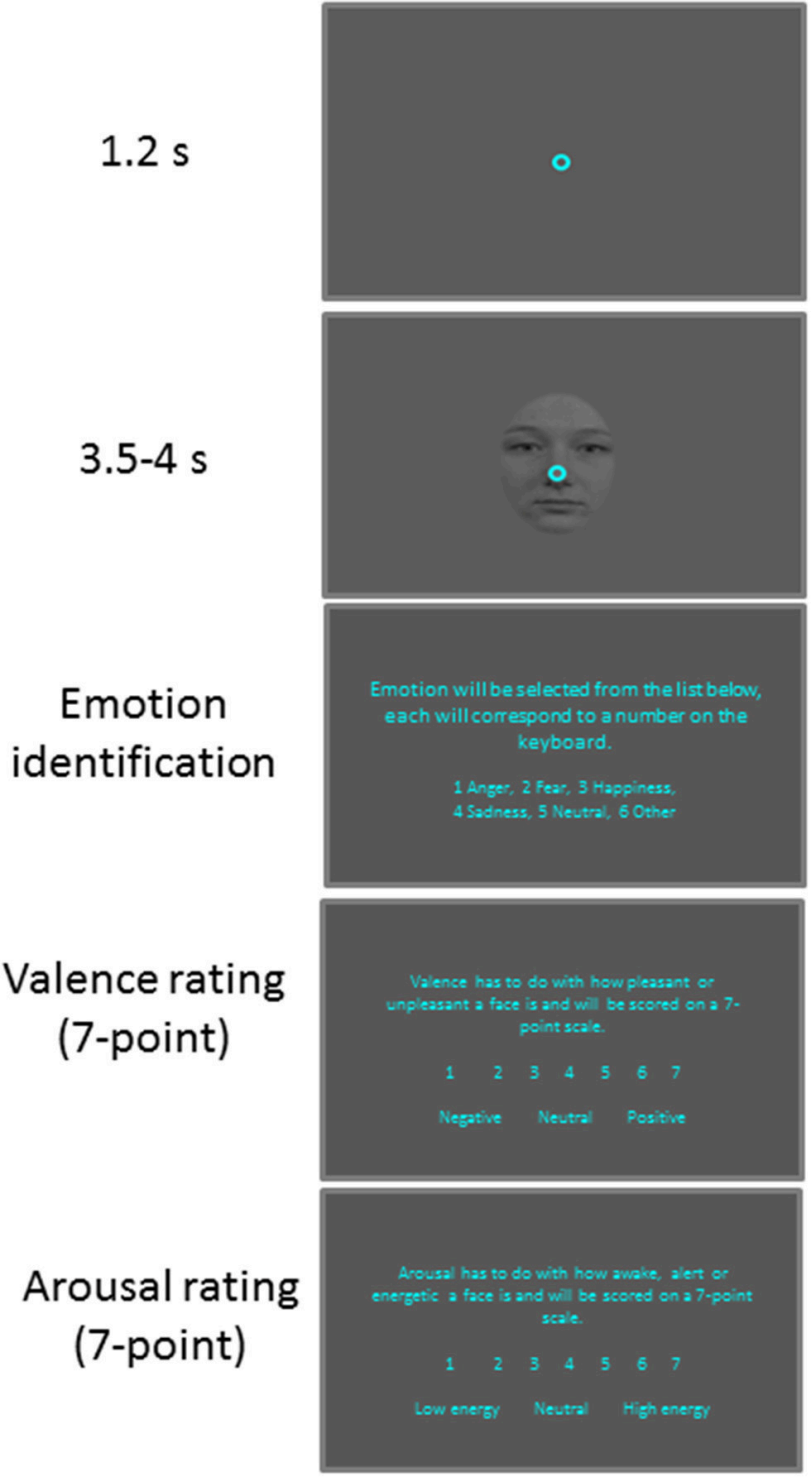
Behavioral paradigm. Each trial began with a central fixation point on a gray background. After a delay, a face stimulus was presented and after a random delay the central fixation point and a face stimulus disappeared and participants were required to answer three questions about the face presented.

The goal of this study is to investigate trial-by-trial fluctuations in sympathetic and parasympathetic modulation of pupil size. To index sympathetic and parasympathetic activity, HR and GSR were recorded concurrently with pupil size. Emotional face stimuli were used to induce arousal fluctuation because they are often used to evoke emotional arousal [e.g., (31–33)]. Participants maintained central fixation during the trial, following the presentation of different emotional face stimuli with carefully controlled low-level visual properties (Figure 1). Considering the common sympathetic control of GSR and pupil size, and common parasympathetic control of HR and pupil size, we hypothesize that activation of the parasympathetic system should decrease HR and pupil size, and activation of the sympathetic system should increase GSR and pupil size, together predicting positive correlations between pupil size and HR or GSR. Moreover, because baseline pupil size and stimulus-evoked (referred to as task-evoked) pupil dilations are thought to reveal different neural processes [e.g., (8, 10, 12)], the epochs before and after face presentation were analyzed separately, to examine the correlations both in baseline and task-evoked responses.

## Materials and methods

### Participants

All experimental procedures were reviewed and approved by the Human Research Ethics Board of Queen's University and were in accordance with the principles of the Canadian Tri-Council Policy Statement (TCPS-2 2014) on Ethical Conduct for Research Involving Humans, and the Declaration of Helsinki (34). Thirty participants (sixteen female) ranging between 18 and 24 years of age (*M* = 21.76, *SD* = 1.56) were recruited for this study. All participants had normal or corrected to normal vision, were naïve to the purpose of the experiment. Participants gave written informed consent and were compensated for their participation. Recruitment was limited to Caucasian participants because the face stimuli used in this experiment were all from Caucasian models.

### Recording and apparatus

Eye movements, pupil size, heart rate and skin conductance were recorded throughout the experiment. A video-based eye tracker (Eyelink-1000 binocular-arm, SR Research, Osgoode, ON, Canada), was used to measure eye position and pupil size with binocular recording at a sampling rate of 500 Hz (left eye was used). Eye position was tracked in order to ensure that participants maintained visual fixation on a point at the center of the screen throughout the trial. Heart rate was measured using a simple photo-sensor digital heart rate monitor that outputs a binary value based on blood flow through the ear (Grove Ear clip heart Beat Sensor). Skin conductance was measured using a galvanic skin conductance sensor (Q-S222 Galvanic Skin Response Sensor, Qubit Systems Inc., Kingston, ON, Canada), and the sensor monitored skin conductivity between two disposable tab electrodes attached to the index and middle fingers of the left hand. We used a 5 uSiemens range, which yielded a resolution of 0.0012 uS, and manually adjusted to an optimal range for each participant during the setup period, prior to the experiment. Both HR and GSR were recorded through an Arduino Uno digital acquisition board (https://www.arduino.cc) at a rate of 210 Hz with 10-bit resolution. Through a serial connection to the Arduino, the biometric recordings were controlled by the Experiment Builder program running the experiment. The biometric recordings could therefore be initiated and terminated by the same program controlling the display, and event marker codes could be placed in the biometric recordings at precise timings, allowing us to precisely align our trial stimulus events to the HR and GSR recordings. For convenience of comparison, all recordings were then interpolated to a 1 ms resolution using a spline interpolation for pupil area and GSR, and a linear interpolation for heart rate. Stimuli were presented on a 17-inch LCD monitor at a screen resolution of 1,280 × 1,024 pixels (60 Hz refresh rate), subtending a viewing angle of 32° × 26°, and the distance from the eyes to the monitor was set at 60 cm. Pupil area values recorded from the eye tracker were transformed to actual pupil size in diameter following previously described methods suggested by the Eyelink company (35, 36). Briefly, laser-printed dots between 2.0 and 6.0 mm in diameter were printed (false pupils), and placed at approximately the same position as the participants' pupil position during data recording. Eyelink pupil values from false pupils were used to transform Eyelink pupil values recorded from real participants to actual pupil diameter simply using a linear interpolation after taking the square root of all pupil area data. According to Eyelink, measurement error is below 1% with under 0.2% error for 3 mm or greater.

### Behavioral task

Participants were seated in a dark room and the experiment consisted of 6 practice trials followed by 100 trials. Each trial (Figure 1) began with the appearance of a central fixation point (FP) (0.6° diameter, 11 cd/m^2^) on a gray background (11 cd/m^2^). After 1,200 ms of central fixation, a centered facial stimulus (3° × 4°, 11 cd/m^2^) with a central FP appeared for 3,500–4,000 ms. This was followed by three questions presented on the visual screen. First, the participants were asked to identify the emotion expressed by keying in a number on a keyboard attributed to one of six options: anger, happiness, fear, sadness, neutral, and other. Participants were then asked to rate the degree of arousal and valence of the stimuli using seven-point scales (17). When rating arousal, 1 indicated a low and 7 indicated a high degree of arousal. When rating valence, 1 indicated an unpleasant stimulus whereas, 7 indicated a pleasant stimulus, with 4 representing a neutral value. The next trial commenced after an inter-trial interval of 3–4 s.

### Stimuli

Adult facial stimuli were selected from the Radboud Faces Database, which had been validated with respect to expression recognition, clarity, genuiness, attractiveness and valence (37) and used previously in our lab (38, 39). Images of 20 (10 male and 10 female) front facing, adult models expressing anger, happiness, fear, and sadness in addition to a neutral expression were incorporated into the fixation task. Images of separate models were used in the initial practice phase of the task. Oval face masks, previously used to isolate the face and eliminate distractions such as hair (40–42) were applied to all faces using Adobe Photoshop Creative Cloud 2015.5 (Adobe Systems Inc., San Jose, CA). Following our previous method (38, 39), after oval masking, Radboud face images were grayscale and adjusted to match the background luminance. They were aligned such that the nose of each image appeared at the FP location. Face stimuli were then filtered through the SHINE MATLAB toolbox to the normalize luminance, visual contrast and spatial frequency of facial images (43). Therefore, luminance, visual contrast and spatial frequency were controlled across all facial stimuli, and the overall luminance level remained unchanged during the trial.

### Data analysis

To maintain an accurate measure of pupil size, trials with an eye position deviation of more than 2° from the central FP or with detected saccades (>2°) during the required period of central fixation were excluded from analysis. Following the literature, a linear interpolation was performed using pre- and post-blink pupil values to replace pupil values during a detected blink (10, 44, 45). Trials were discarded when two eye blinks occurred within a time interval of 500 ms. The above criteria resulted in the removal of 11.0% of trials. Four participants were excluded from GSR analyses due to recording errors, and one participant was excluded from HR analyses due to recording errors. In addition, 48.7% of trials were removed from GSR analysis due to reading values beyond the range of the recording system (5 μsigmen). Note that, because there were at least 10 valid trials in a given condition for all included participants for each analysis (except for valence analysis, which only required 5 trials), the number of included participants was different among different analyses. Heart rate for each participant was analyzed by identifying the onset of each peak, representing a beat on our photo sensor. The timing of all beats for all trials were then overlaid to generate a raster plot of beats. This was then smoothed using a rectangular zero-phase (filtered both forward and reverse) filter of 100 ms to produce a continuous beat-per-minute trace for each trial type.

The raw values averaging from 1,000 ms before to the onset of the face presentation in pupil size, HR, and GSR were used to investigate the correlation among these measurements before the face presentation (referred to as pre-stimulus epoch). To investigate the task-evoked responses, baseline-correction procedure was used. For pupil size, a baseline pupil value for each trial was determined by averaging pupil size from 500 ms before to the onset of the face presentation, as used previously (36, 46). Pupil values were subtracted from this baseline value, and the mean change (from baseline) in an epoch from 500 to 3,000 ms after picture onset was used to indicate task-evoked pupil responses. Following previous research on heart rate and skin conductance analyses (47), baseline (averaging from 1,000 ms before to the face appearance) was subtracted from the GSR and HR values. For heart rate, the mean change (from baseline) in an epoch from 500 to 3,000 ms after stimulus onset was used. For skin conductance, the maximum change between 500 and 3,000 ms after face onset was computed with a log transform (log [GSR]). Note that outlier values beyond ±2.5 standard deviation were also excluded from analysis. To examine the hypothesis that pupil size should correlate with both HR and GSR with larger pupil size for higher HR or GSR, we performed correlational analyses and a one-tailed student *t*-test except where indicated. Bayesian *t*-test was also performed to inform statistical significance for pairwise comparisons, with a scale factor *r* = 0.707 (48). Moreover, Cohen's *d*, where appropriate, was calculated to estimate the effect size (49). One way repeated-measure ANOVA with Bonferroni-corrected *post hoc* comparisons was performed to assess the effect of emotion on behavioral responses, pupil size, heart rate, and skin conductance values.

To analyze, on a trial-by-trial basis, whether subjective arousal value for each face stimulus can be predicted by task-evoked responses of pupil size, HR, and GSR during face viewing, we employed a logistic regression approach. More specifically, we performed an individual logistic regression for each participant to estimate the predictive value of each task-evoked response to the arousal rating for each individual face stimulus presentation. The normalized beta-values (beta-values/standard errors of beta-values) from these individual logistic regressions were then subjected to two-tailed *t*-tests at the group-level to assess whether the beta-values were reliably different from zero.

Multiple regression analysis was used to determine if and how HR and GSR influenced pupil size. To estimate the contribution of HR and GSR on pupil size before and after face stimulus presentation on each trial, we performed the multiple regression analysis in the two epochs on a trial-by-trial basis separately for all participants using HR (Equation 1), GSR (Equation 2), or HR+GSR (Equation 3) as independent variables in the analysis. We then compared adjusted R-square values derived from the model in all participants using two-tailed student *t*-test to evaluate at the group-level whether combined HR+GSR explains significantly more variance of pupil size than the HR- or GSR- alone condition. If HR and GSR uniquely contribute to pupil size, adjusted R-square values of the combined conditions (HR+GSR) should be larger than the HR- or GSR- alone condition.

$$\begin{array}{l} \text{Pupil size}={\text{a}}^{*}\text{HR}+\text{b}\\ \text{ Pupil size}={\text{a}}^{*}\text{GSR}+\text{b}\\ \text{Pupil size}={\text{a}}^{*}\text{HR}+{\text{b}}^{*}\text{GSR}+\text{c} \end{array}$$

a, b, c were constant linear weights generated by the model.

## Results

### Behavioral performance in recognizing facial emotions

**Figure 2:**
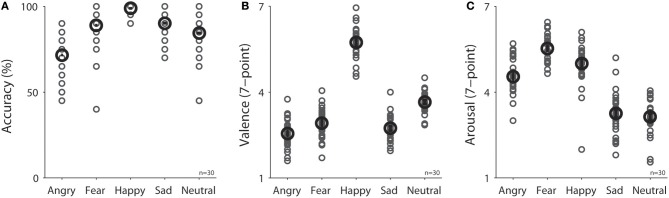
Task performance in different emotional face conditions (*N* = 30) on **(A)** accuracy in emotion recognition, **(B)** valence rating, and **(C)** arousal rating. The black bold-circle represents the mean value across participants. The error-bar represents ±standard error across participants. The gray small-circle represents mean value for each participant. n, number of participants.

Participants were engaged during the experiment because they performed the task accurately with correct responses made for 75, 90, 99, 90, and 85% of trials in the angry, fear, happy, sad, and neutral condition, respectively [Figure 2A, *F*_(4, 116)_ = 27.33, *p* < 0.001, *N* = 30]. Mean valence ratings (7-point scale) were 2.55, 2.92, 5.74, 2.73, and 3.65 in the angry, fear, happy, sad, and neutral condition, respectively [Figure 2B, *F*_(4, 116)_ = 281.27, *p* < 0.001, *N* = 30], and as expected, valence values were lower for negative emotions than positive or neutral emotions (all *p*s < 0.05). Mean arousal ratings (7-point scale) were 4.54, 5.53, 5.00, 3.25, and 3.14 in the angry, fear, happy, sad, and neutral condition, respectively [Figure 2C, *F*_(4, 116)_ = 107.33, *p* < 0.001, *N* = 30].

**Figure 3:**
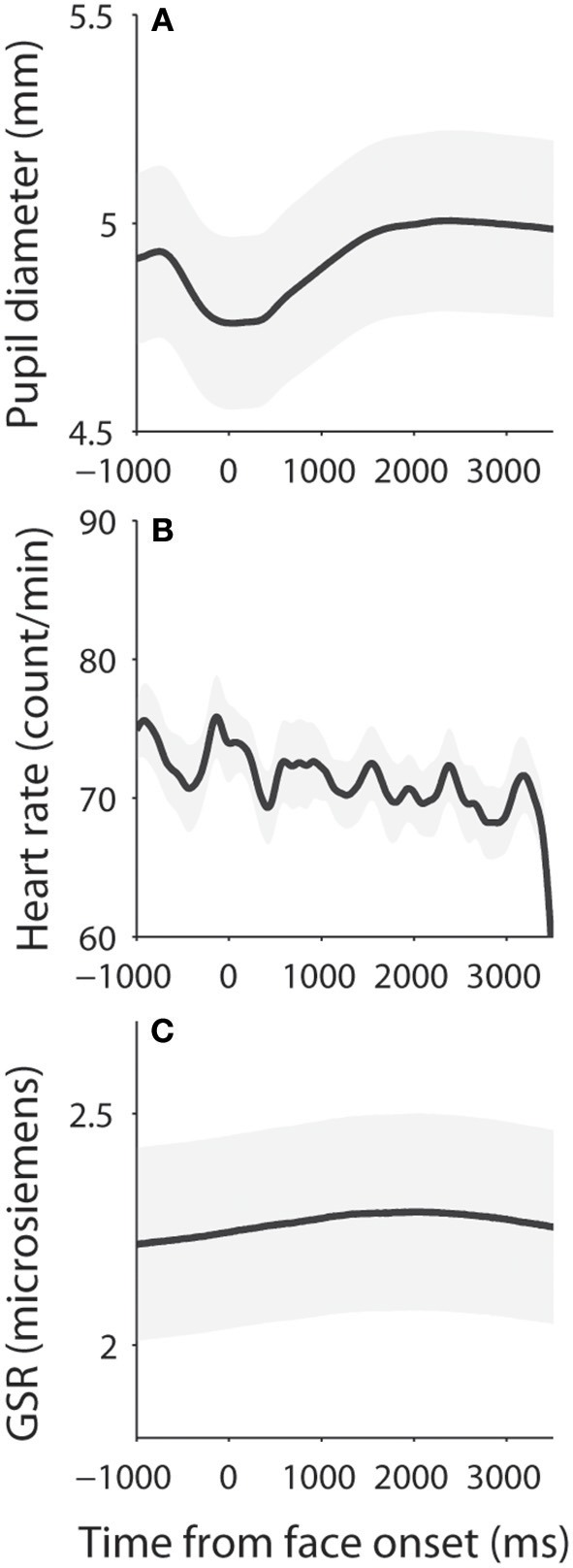
Dynamics of pupil size, heart rate, and skin conductance responses. **(A)** Pupil diameter following the presentation of face stimuli. **(B)** Heart rate following the presentation of face stimuli. **(C)** Galvanic skin response following the presentation of face stimuli. The shaded gray regions surrounding the response represent ± standard error range (across participants). GSR, galvanic skin response.

Dynamics of measured responses are shown in Figure 3. In general, pupil size decreased before face presentation and pupil dilation was observed after the face presentation (Figure 3A). Initial pupil constriction after central fixation, prior to stimulus presentation, has been observed in many studies, including those conducted in our lab. There is no good argument to explain this pupil constriction, but it is possible that this constriction may be associated with the beginning of central fixation or the engagement of attention. The observed pupil dilation was consistent with a recent study which presented affective stimuli centrally while controlling low-level visual properties of the stimuli (50). In addition, heart rate (HR) and GSR were simultaneously recorded (Figures 3B,C) to index activity of the parasympathetic and sympathetic system, respectively. We first examined the relationship between pupil diameter and HR or GSR before face presentation (pre-stimulus epoch), and then examined correlation of the task-evoked responses in pupil size, HR, and GSR after face presentation.

### Pupil diameter correlated with heart rate and skin conductance before face presentation

**Figure 4:**
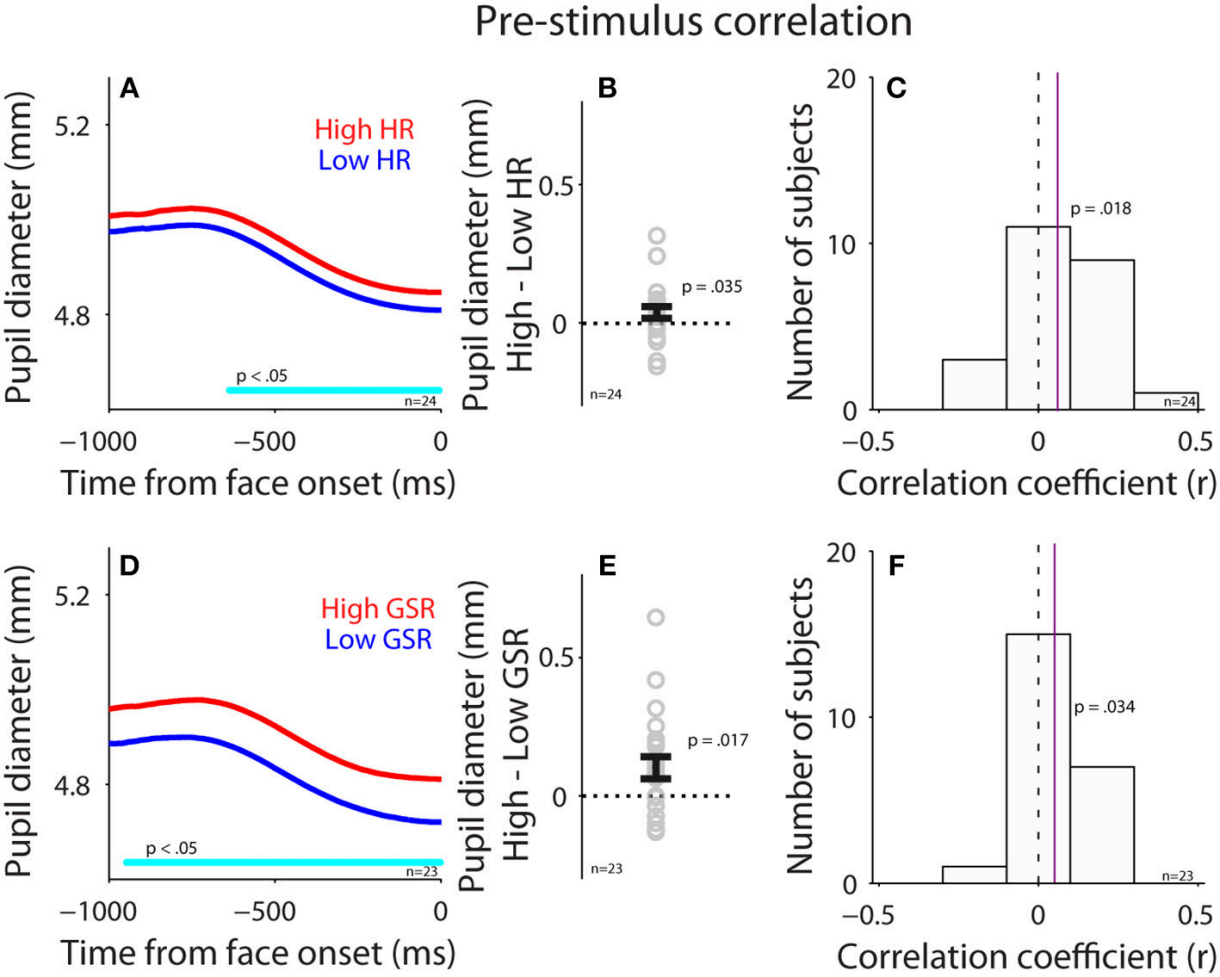
Correlation between pupil diameter and HR or GSR before face presentation. **(A)** Pupil diameter following the presentation of face stimuli in higher or lower HR (*N* = 24). **(B)** Differences in pupil diameter between higher and lower HR conditions for each individual subject. **(C)** Distribution of correlation coefficients for the relationship between pupil diameter and HR for all subjects. **(D)** Pupil diameter following the presentation of face stimuli in higher or lower GSR (*N* = 23). **(E)** Differences in pupil diameter between higher and lower GSR conditions for each individual subject. **(F)** Distribution of correlation coefficients for the relationship between pupil diameter and GSR for all subjects. In **(A,D)**, the cyan bar on X-axis indicates the time line at which differences between the two conditions were statistically significant (*p* < 0.05). In **(B,E)**, the error-bar represents mean ± standard error across participants. In **(C,F)**, the vertical dotted line represents a zero value of the correlation coefficient (*r* = 0), and the vertical purple line represents the median value of the correlation coefficient. HR, heart rate; GSR, galvanic skin response; n, number of participants.

To investigate the influence of the parasympathetic system on pupil size before face presentation, we performed a correlation between pupil diameter (raw pupil size) and HR before the presentation of face stimuli (pre-stimulus epoch). Trials were divided into two groups according to HR in the pre-stimulus epoch (median-split), and pupil dynamics between higher and lower heart rate were different (Figure 4A), with larger pupil diameter when HR was higher [mean pupil size diameter of epoch from 500 ms to face onset: high: 4.89, low: 4.85, *t*_(23)_ = 1.85, *p* = 0.035, BF = 0.93, *d* = 0.38, *N* = 24, Figure 4B: high-low]. Figure 4C shows summary histogram of trial-by-trial correlation coefficients for all subjects, showing a positive correlation between HR and pupil diameter [median correlation coefficient: 0.06, *t*_(23)_ = 2.3, *p* = 0.018, BF = 1.92, *d* = 0.64, one-tailed paired *t*-test of R values against zeros], suggesting a correlation between heart rate and pupil diameter before face presentation. These results were consistent with the hypothesis that an increase in parasympathetic activity resulted in decreased heart rate and pupil size.

Correlation between GSR and pupil diameter was also observed before face presentation (Figures 4D–F). Trials were divided into two groups according to GSR during the pre-stimulus epoch, and pupil dynamics between higher and lower GSR (median-split) in the pre-stimulus epoch were different (Figure 4D), with significantly larger pupil diameter in the higher GSR condition, compared to the lower GSR condition [mean pupil size of epoch from 500 ms to face onset: high: 4.85, low: 4.76, *t*_(22)_ = 2.26, *p* = 0.017, BF = 1.79, *d* = 0.47, *N* = 23, Figure 4E: high-low]. Figure 4F shows summary histograms of trial-by-trial correlation coefficients for all subjects, showing a positive correlation between GSR and pupil diameter [median correlation coefficient: 0.05, *t*_(22)_ = 1.8, *p* = 0.034, BF = 0.87, *d* = 0.53]. Consistent with the hypothesis, these results suggest that activation of the sympathetic pathway caused an increase in GSR and pupil size. Overall, these results suggest a small but reliable correlation between pupil size and HR or GSR. Note that BF values were not decisive in these statistical tests, therefore the results should still be explained with caution.

### No modulation of pupil size, HR, GSR by facial emotional stimuli

**Figure 5:**
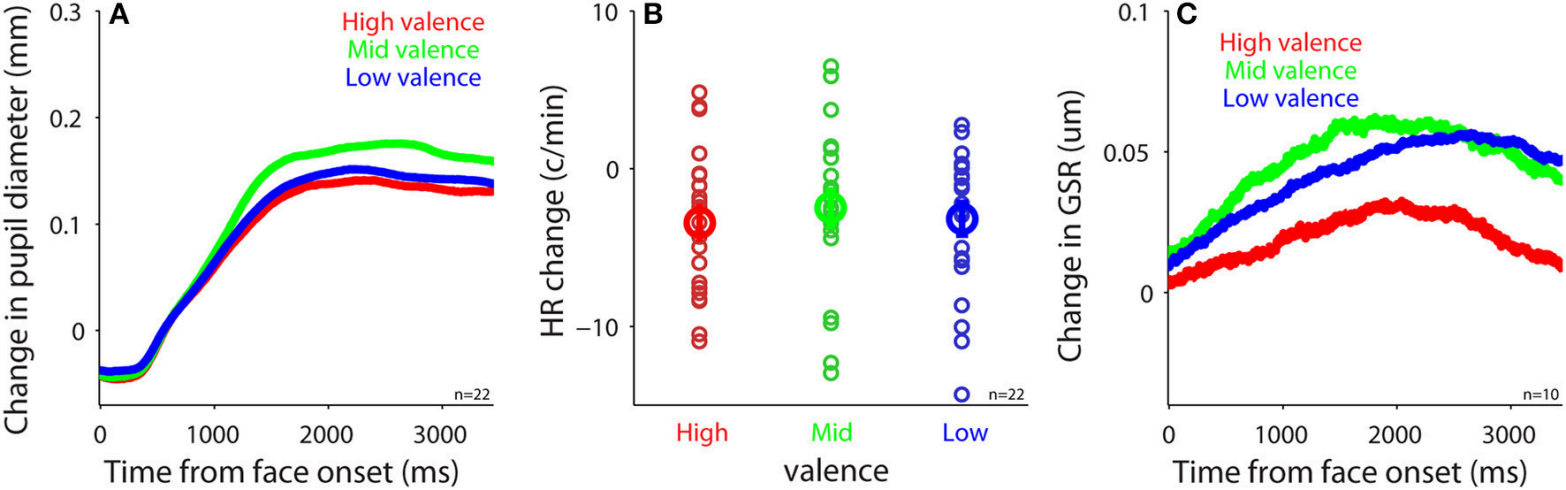
Task-evoked responses after face presentation. **(A)** Change in pupil size following the presentation of face stimuli in different valence conditions (*N* = 22). **(B)** Heart rate change after face presentation in different valence conditions (*N* = 22). **(C)** Change in GSR after face presentation in different valence conditions (*N* = 10). In **(B)**, the bold-circle represents the mean value across participants. The error-bar represents ± standard error across participants. The colored small-circle represents mean value for each participant. HR, heart rate; GSR, galvanic skin response; n, number of participants.

To investigate the modulation of task-evoked responses (see Methods) by emotional valence, we separated trials into three emotion categories (positive: 5–7 valence value; neutral: 4 valence value; negative: 1–3 valence value) according to the subjective valence ratings. Presentation of face stimuli evoked pupil dilation regardless of valence level (Figure 5A), which was similar to other studies (e.g., 50). However, in contrast to other studies using images (47, 51–53), emotional valence did not modulate evoked pupil responses, with mean pupil responses being 0.11, 0.14, and 0.12 in the positive, neutral, and negative conditions, respectively [*F*_(2, 42)_ = 2.22, *p* = 0.12, all *p*s > 0.23, *N* = 22]. Although presentation of face stimuli generally decreased HR and increased GSR responses (Figures 5B,C), unlike other studies (17, 47, 54, 55), task-evoked responses in HR and GSR were not modulated by emotional valence, with mean HR change being −3.42, −2.5, and −3.19 in the positive, neutral, and negative conditions, respectively [Figure 5B, *F*_(2, 42)_ = 0.27, *p* = 0.76, all *p*s > 0.9, *N* = 22], and mean GSR change was 0.049, 0.072, and 0.063 in the positive, neutral, and negative conditions, respectively [Figure 5C, *F*_(2, 18)_ = 0.43, *p* = 0.61, all *p*s > 0.9, *N* = 10]. Note that there were only 10 participants included in GSR analysis, therefore the non-significant results could be due to a weak statistical power. Previous research has shown no differences in pupil responses evoked by emotional stimuli among positive, neutral, and negative emotions when the intensity of emotions is low (56) [similar results in GSR and HR (54)]. It is thus possible that the intensity of emotion in our stimuli was too low to produce a pronounced valence modulation because we specifically controlled low-level visual properties across all face stimuli. Notably, research has shown differences between explicit and implicit emotional processing (57–59), and weaker emotional modulation when the executive control is involved (60). Therefore, it is also possible that our explicit task requirement for emotion identification and valence and arousal rating automatically engaged the executive network, which greatly interrupted normal emotional face processing, resulting in weak emotional effects. Future research is required to address these possibilities.

### Correlation between task-evoked pupil responses and heart rate and skin conductance

**Figure 6:**
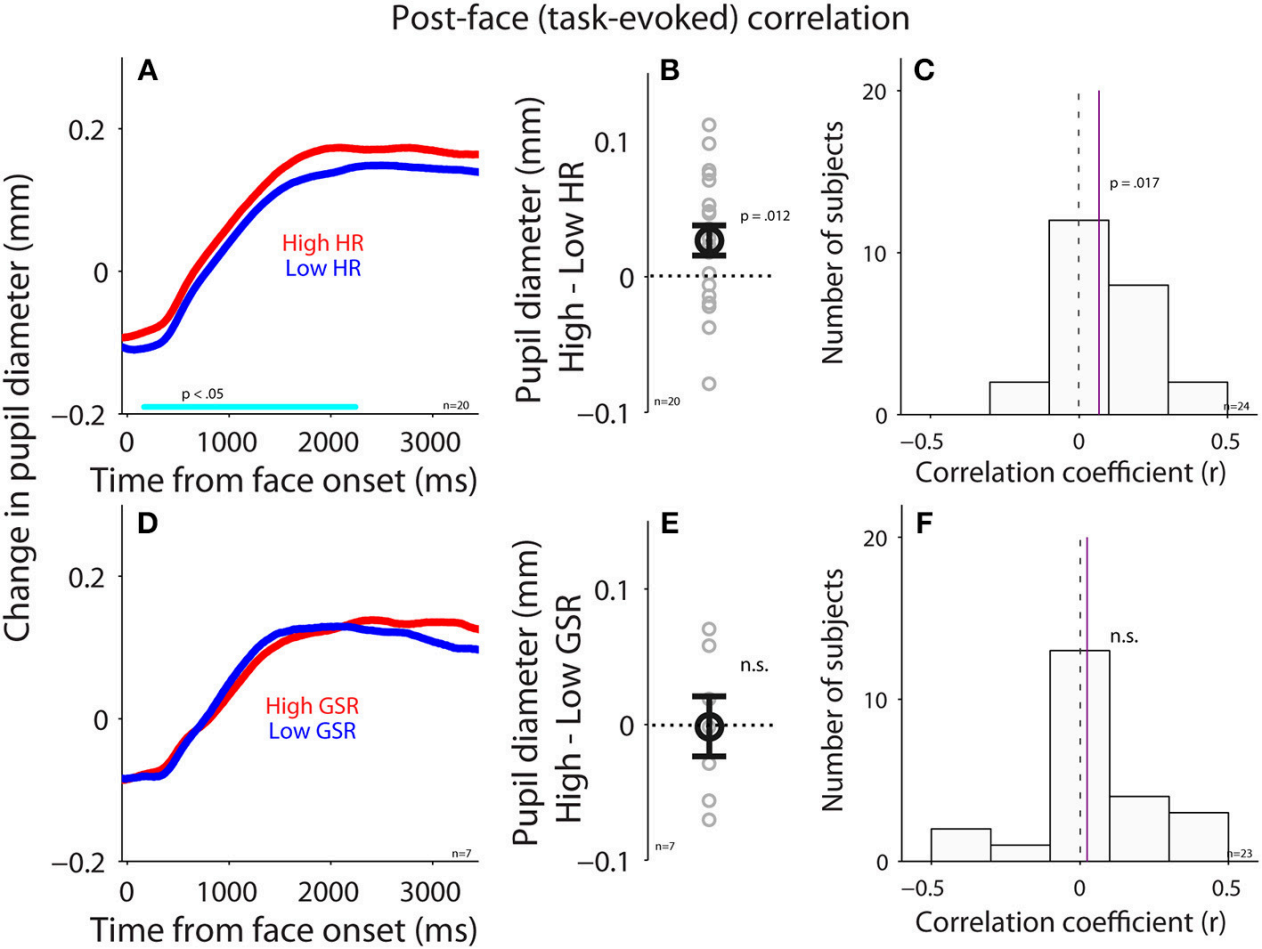
Correlation between task-evoked pupil response and HR or GSR after face presentation. **(A)** Task-evoked pupil responses following the presentation of face stimuli in higher or lower HR (*N* = 20). **(B)** Differences in task-evoked pupil responses between higher and lower HR conditions for each individual subject. **(C)** Distribution of correlation coefficients for the relationship between task-evoked pupil responses and HR for all subjects (*N* = 29). **(D)** Task-evoked pupil responses following the presentation of face stimuli in higher or lower GSR (*N* = 7). **(E)** Differences in task-evoked pupil responses between higher and lower GSR conditions for each individual subject. **(F)** Distribution of correlation coefficients for the relationship between task-evoked pupil responses and GSR for all subjects (*N* = 23). In **(A,D)**, the cyan bar on X-axis indicates the time line at which differences between the two conditions were statistically significant (*p* < 0.05). In **(B,E)**, the bold-circle represents the mean value across participants. The error-bar represents ± standard error across participants. The colored small-circle represents mean value for each participant. In **(C,F)**, the vertical dotted line represents a zero value of the correlation coefficient (*r* = 0), and the vertical purple line represents the median value of the correlation coefficient. HR, heart rate; GSR, galvanic skin response; n.s., not statistically significant; n, number of participants.

To examine the parasympathetic and sympathetic modulation on pupil size after face presentation, we performed correlations between task-evoked pupil responses and task-evoked HR or GSR (see Methods). Trials were divided into two groups according to task-evoked HR after face presentation (median-split), and pupil size between higher and lower HR were different (Figure 6A), with significantly larger pupil dilation when HR was higher [mean pupil size: high: 0.13, low: 0.098, *t*_(19)_ = 2.47, *p* = 0.012, *d* = 0.55, BF = 2.57, *N* = 20, Figure 6B: high-low]. Figure 6C shows summary histogram of trial-by-trial correlation coefficients for all subjects, showing a positive correlation between task-evoked pupil and HR responses [median correlation coefficient: 0.067, *t*_(23)_ = 2.3, *p* = 0.017, BF = 1.91, *d* = 0.64, *N* = 24], suggesting a correlation in task-evoked responses between heart rate and pupil size. In contrast, task-evoked pupil responses did not correlate with GSR after face presentation. Trials were divided into two groups according to task-evoked GSR after face presentation (median-split), and pupil dynamics between higher and lower GSR were not different (Figure 6D), with similar pupil dilations between two conditions [mean pupil size: high: 0.091, low: 0.092, *t*_(6)_ = 0.07, *p* = 0.45, BF = 0.35, *d* = 0.03, *N* = 7, Figure 6E: high-low]. Figure 6F shows summary histogram of trial-by-trial correlation coefficients for all subjects, showing again no correlations between task-evoked pupil and GSR responses [median correlation coefficient: 0.025, *t*_(22)_ = 0.63, *p* = 0.27, BF = 0.26, *d* = 0.18, *N* = 23]. Note that the number of subjects was different between these two analyses because median-split in the first analysis resulted in two conditions, and subjects required a sufficient number of trials in both conditions to be included in the analysis, resulting in fewer viable subjects.

### GSR during face viewing predicts subsequent arousal rating

**Figure 7:**
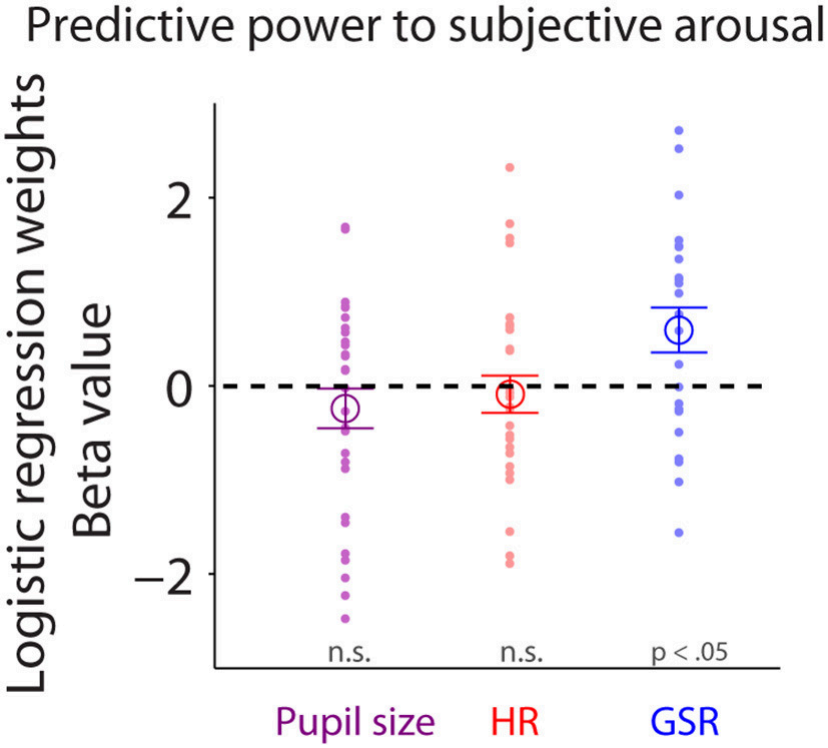
Trial-by-trial relationship between subjective arousal and task-evoked pupil size, HR, and GSR. Individual normalized beta values from logistic regression analyses for pupil size, HR, and GSR on arousal rating (*N* = 23). The large-circle represents the mean value across participants. The error-bar represents ± standard error across participants. The colored small-dot represents mean value for each participant. HR, heart rate; GSR, galvanic skin response; n.s., not statistically significant.

To examine trial-by-trial relationships between subjective arousal of emotional faces and task-evoked responses in pupil size, HR, or GSR, we performed logistic regression (see Methods). Task-evoked pupil responses during face viewing did not predict trial-by-trial variability of subjective arousal [Figure 7: mean beta-value: −0.24, *t*_(28)_ = −1.14, *p* = 0.27, BF = 0.36, *d* = 0.29, two-tailed paired *t*-test of ß values against zeros]. Responses in HR also failed to predict trial-by-trial variability of subjective arousal [Figure 7: two-tailed paired *t*-test: mean beta-value: −0.089, *t*_(27)_ = −0.452, *p* = 0.65, BF = 0.22, *d* = 0.12]. Yet, GSR responses during face viewing reliably predicted trial-by-trial variability of subjective arousal [Figure 7: two-tailed paired *t*-test: mean beta-value: 0.59, *t*_(22)_ = 2.47, *p* = 0.022, BF = 2.58, *d* = 0.72], suggesting that task-evoked GSR can predict subjective arousal in the context of emotional face viewing.

### Modeling pupil size using HR and GSR

**Figure 8:**
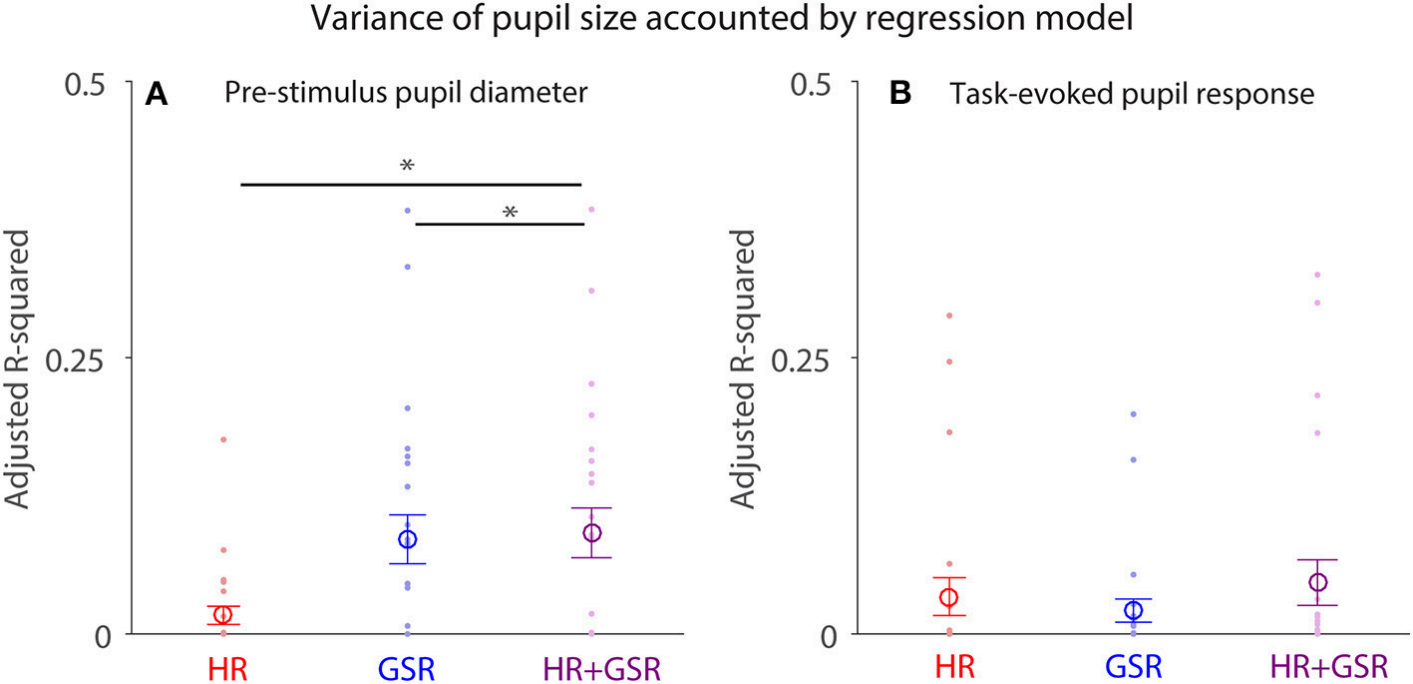
Contribution of HR and GSR on trial-by-trial variation in pupil size before (pre-stimulus) and after (task-evoked) face presentation**. (A)** Adjusted R-square values derived from the regression model using baseline HR+GSR, HR, or GSR as independent variables to explain trial-by-trial variation in pupil diameter before face presentation (baseline). **(B)** Adjusted R-square values derived from the regression model using task-evoked HR+GSR, HR, or GSR as independent variables to explain trial-by-trial variation in task-evoked pupil size after face presentation. The large-circle represents the mean value across participants. The error-bar represents ± standard error across participants. The colored small-dot represents mean value for each participant. HR, heart rate; GSR, galvanic skin response. *indicates differences are statistically significant.

Pupil size is controlled by the activity of the parasympathetic and sympathetic systems (7), therefore HR and GSR should influence a trial-by-trial fluctuation of pupil size differently. To test this hypothesis, we performed a multiple regression analysis, and used HR (Equation 1), GSR (Equation 2), or HR+GSR (Equation 3) on a trial-by-trial basis as independent variables to account for trial-by-trial pupil size fluctuation in both pre-stimulus and post-face (task-evoked responses) epochs (*N* = 24). Although trial-by-trial pupil size fluctuation in the pre-stimulus epoch explained by the model was generally small (Figure 8A), with the mean variance (adjusted R-squared) being 0.017, 0.086, and 0.092 in the HR, GSR, and HR+GSR condition in the pre-stimulus epoch, respectively, adjusted R-squared values were significantly higher in the HR+GSR condition than in the HR- or GSR- alone condition [two-tailed paired *t*-test: HR+GSR and HR: *t*_(23)_ = 4.53, *p* = 0.00015, BF = 188.00, *d* = 0.92; HR+GSR and GSR: *t*_(23)_ = 4.18, *p* = 0.00036, BF = 86.42, *d* = 0.85]. Similar, but not significant, patterns were observed in the post-face epoch (Figure 8B), with the mean variance (adjusted R-squared) being 0.034, 0.021, and 0.047 in the HR, GSR, HR+GSR condition, respectively [two-tailed paired *t*-test: HR+GSR and HR: *t*_(23)_ = 1.34, *p* = 0.19, BF = 0.47, *d* = 0.35; HR+GSR and GSR: *t*_(23)_ = 1.38, *p* = 0.18, BF = 0.50, *d* = 0.34]. These results suggest that both HR and GSR uniquely accounted for some fluctuations of pupil size on a trial-by-trial basis in the pre-stimulus epoch, arguably mediated by the parasympathetic and sympathetic system, respectively.

## Discussion

Pupil size is becoming an increasingly popular index of arousal and cognitive function, largely due to the popularity of the video-based eye-tracking system with automated pupillometry. Here, we directly examined the relationships between pupil size and parasympathetic and sympathetic activity, through simultaneous recordings of pupil size, heart rate (HR), and galvanic skin response (GSR) during an emotional face recognition task. Pupil diameter on a trial-by-trial basis positively correlated with HR and GSR before face presentation: trials with larger pupil diameter prior to face presentation were accompanied by higher HR and GSR (Figure 4). Although trial-by-trial correlation between task-evoked pupil responses and GSR after face presentation was diminished (Figure 6), trial-by-trial variations in GSR after face presentation reliably predicted subsequent subjective arousal rating (Figure 7). Moreover, both HR and GSR, as an index of parasympathetic and sympathetic activity, uniquely accounted for the variance of pupil size fluctuation on a trial-by-trial basis (Figure 8). Together, our results suggest that pupil size correlated with measures of both the parasympathetic and sympathetic systems.

### Autonomic control of pupil size, heart rate, and skin conductance

Although HR, GSR and pupil size all associate with activity of the autonomic nervous system, the underlying neural substrate mediating each index is very different. Autonomic control of cardiac activity begins in the medulla. The nucleus of the solitary tract inhibits the sympathetic rostral ventrolateral medulla and activates the parasympathetic dorsal vagal nucleus (61) and nucleus ambiguus (62) which contribute to the vagal nerve. Neurons in the sympathetic rostral ventrolateral medulla project to preganglionic spinal cord neurons. Post-ganglionic sympathetic and parasympathetic neurons in the stellate (63) and cardiac ganglia (61), respectively, innervate the heart. Although predominantly controlled by the parasympathetic system, HR is also modulated by the sympathetic system. Sympathetic pre-ganglionic neurons in the spinal cord also innervate the adrenal medulla, a modified sympathetic prevertebral ganglion, stimulating the release of epinephrine and norepinephrine into the blood stream, which then travels to the heart (64). Sympathetic control of sweat gland activity begins in the preoptic sweat nucleus of the hypothalamus, which projects to preganglionic neurons in the intermediolateral spinal cord (65). These neurons travel through the ventral root of the spinal cord to innervate postganglionic sympathetic neurons in the paravertebral sympathetic chain ganglia. These neurons project to sympathetic terminals surrounding sweat glands (66). Therefore, increases in sympathetic activity produce corresponding increases in GSR (5).

Pupil size is controlled by the balanced activity of the sympathetic and parasympathetic nervous system, with parasympathetic and sympathetic innervation of the pupillae sphincter and dilator pupillae muscles of the iris, respectively (6, 7). Parasympathetic innervation of the pupillae sphincter comes from preganglionic neurons in the Edinger-Westphal nucleus in the midbrain [reviewed in (67)]. Preganglion neurons of the Edinger-Westphal nucleus project to postganglionic pupilloconstrictor neurons in the ciliary ganglion, which in turn control constrictor pupillae muscles directly through a short projection (68). In the sympathetic pathway, preganglionic sympathetic neurons located in the ciliospinal center of Budge, the C8-T2 segments of the spinal cord, project to sympathetic chain ganglia and travel to the superior cervical ganglia through the sympathetic trunk to the superior cervical ganglion (67). Here, post-ganglionic sympathetic neurons project to the dilatory pupillae via long and short ciliary nerves (69).

Given the abovementioned pathways, pupil size should correlate with HR and GSR. Consistently, we found a positive trial-by-trial correlation between pupil diameter and HR or GSR before face presentation, with larger pupil diameter observed on trials with higher HR or larger GSR responses (Figure 4). Since HR is also modulated by the sympathetic pathway, the observed correlations between HR and pupil size can also be partly attributed by the sympathetic pathway. After face presentation, however, this correlation was diminished particularly between pupil size and GSR (Figure 6). The diminished correlation after face presentation could be due to low intensity of emotional face stimuli resulting from the control of low-level visual properties across stimuli including luminance, visual contrast, and spatial frequency. As a result, there were no differences in valence modulation of pupil size, HR, or GSR (Figure 5). The uncontrolled intensity of different emotions and different task requirements for emotional image stimuli may also explain a degree of inconsistency observed in the valence modulation of pupil size, HR, or GSR in the literature (17, 47, 52, 53, 55, 56, 70–73). Moreover, the inconsistency of the valence modulation with imaging viewing could also be attributed to inadequate control of low-level visual properties across stimuli and evoked eye movements across conditions. It is also interesting to note that this valence modulation of pupil dilation can be evoked with emotional written words, and previous studies have shown that larger pupil dilation is evoked by negative words than the neutral or positive words (71–75).

The regression model results suggested that HR and GSR accounted uniquely, arguably mediated separately by the parasympathetic and sympathetic systems, to trial-by-trial pupil size fluctuation because the combined HR and GSR conditions explained more pupil size variance than the HR- or GSR- alone condition (Figure 8). Notably, ~10% of variance in pupil size was explained by HR and GSR in the model. These results could imply that pupil size is more sensitive to autonomic arousal than HR and GSR, because, as described previously, it more directly links to the autonomic nervous system than other indices. It is also possible that pupil size is influenced by other factors which have not been identified. Together, it is important to investigate the influence of the emotional intensity on different autonomic indexes to study the emotional arousal in the future.

### Locus ceruleus-norepinephrine (LC-NE) account for pupil size fluctuation

The LC-NE system has been associated with many functions arguably via arousal mechanisms (76–80), and pupil size variation is regularly linked to the LC-NE system (8). Relationship between pupil size and LC activity has been demonstrated in studies recording neuronal activity in behaving animals (81–85). In humans, drugs assumed to alter LC activity also change pupil size (86), and pupil diameter correlates with LC activation in fMRI study (87, 88). Moreover, drugs that alter arousal state interrupt functional connectivity of the arousal circuit mediated through the LC (89). Notably, there are two modes of LC activity that have been described: tonic and phasic mode, both of which have important behavioral relevance (8) and are thought to affect baseline pupil size and task-evoked pupil dilations, respectively (10, 12). Our results showing a stronger correlation between pupil diameter and HR or GSR before face presentation (baseline pupil size) suggest that tonic LC activity is particularly correlated with the sympathetic and parasympathetic activity observed in the current study. Notably, some other areas such as amygdala and limbic structures may also play an important role in the relation between emotional processing and pupil size [e.g., (90–92)] and therefore possibly contributed to the correlation observed in the current study.

### Other influence of pupil size by low-level visual properties and oculomotor pathway

To fully understand the modulation of pupil size, it is also important to consider other influences on the pupil. Pupil size is also modulated by low-level visual properties in addition to the well-described luminance modulation, and pupil responses to different colors, visual contrast, and spatial frequencies have been observed both in humans and animals (23–26). However, research examining the relationship between pupil size and emotional arousal has mostly only focused on the control of the luminance modulation. Furthermore, eye movements can influence not only the accuracy of pupil size measurement in any video-based camera, but also pupil size itself even if the recording accuracy is maintained through some sorts of calibration. This is because the superior colliculus, a subcortical center for saccadic eye movements (93–95), links to not only shifts of attention and gaze, but also pupil size (30, 96, 97). Weak microstimulation of the SCi (or frontal eye field) evokes pupil dilation without evoking saccadic eye movements (27–29). The superior colliculus-to-pupil control pathway suggests that distinct patterns of eye movements could modulate pupil size differently through the mediated activity in the superior colliculus. In summary, the influences of these factors on pupil size should be carefully considered in the interpretation of any pupil results.

## Conclusion

Pupil size can change independently of changes in luminance, and this trial-by-trial fluctuation in pupil size has largely been attributed to changes in the autonomic arousal level. Here, we showed that pupil size on a trial-by-trial basis particularly before face presentation correlated with both HR and GSR, respectively, indexing activity of parasympathetic and sympathetic branches of the autonomic nervous system. These results suggest that pupil size can be used as an index for the parasympathetic and sympathetic activity on a trial-by-trial basis. Many other factors are also associated with or related to the autonomic system such as blood pressure and glucose level. It is therefore important to record other autonomic indices in addition to pupil size to better understand the modulation of pupil size by the autonomic function.

## Author contributions

C-AW and DM designed research. TB performed research. C-AW and DB contributed unpublished reagents, analytic tools. C-AW and TB analyzed data. C-AW, TB, JH, JC, and DM wrote the paper.

### Conflict of interest statement

The authors declare that the research was conducted in the absence of any commercial or financial relationships that could be construed as a potential conflict of interest.
